# circFTO from M2 macrophage-derived small extracellular vesicles (sEV) enhances NSCLC malignancy by regulation miR-148a-3pPDK4 axis

**DOI:** 10.1007/s00262-024-03634-4

**Published:** 2024-03-30

**Authors:** Qingtao Liu, Pei Xu, Mingming Jin, Lei Wang, Fengqing Hu, Qi Yang, Rui Bi, Haibo Xiao, Lianyong Jiang, Fangbao Ding

**Affiliations:** 1 Department of Cardiothoracic Surgery, School of Medicine, Xinhua Hospital Affiliated Shanghai Jiao Tong University, Shanghai, 200092 People’s Republic of China; 2 Shanghai Key Laboratory of Molecular Imaging, Shanghai University of Medicine and Health Sciences, Shanghai, 201318 People’s Republic of China

**Keywords:** NSCLC, circFTO, miR-148a-3p, PDK4, Small extracellular vesicles (sEV)

## Abstract

**Background:**

Accumulation studies found that tumor-associated macrophages (TAMs) are a predominant cell in tumor microenvironment (TME), which function essentially during tumor progression. By releasing bioactive molecules, including circRNA, small extracellular vesicles (sEV) modulate immune cell functions in the TME, thereby affecting non-small cell lung cancer (NSCLC) progression. Nevertheless, biology functions and molecular mechanisms of M2 macrophage-derived sEV circRNAs in NSCLC are unclear.

**Methods:**

Cellular experiments were conducted to verify the M2 macrophage-derived sEV (M2-EV) roles in NSCLC. Differential circRNA expression in M0 and M2-EV was validated by RNA sequencing. circFTO expression in NSCLC patients and cells was investigated via real-time PCR and FISH. The biological mechanism of circFTO in NSCLC was validated by experiments. Our team isolated sEV from M2 macrophages (M2Ms) and found that M2-EV treatment promoted NSCLC CP, migration, and glycolysis.

**Results:**

High-throughput sequencing found that circFTO was highly enriched in M2-EV. FISH and RT-qPCR confirmed that circFTO expression incremented in NSCLC tissues and cell lines. Clinical studies confirmed that high circFTO expression correlated negatively with NSCLC patient survival. Luciferase reporter analysis confirmed that miR-148a-3p and PDK4 were downstream targets of circFTO. circFTO knockdown inhibited NSCLC cell growth and metastasis in in vivo experiments. Downregulating miR-148a-3p or overexpressing PDK4 restored the malignancy of NSCLC, including proliferation, migration, and aerobic glycolysis after circFTO silencing.

**Conclusion:**

The study found that circFTO from M2-EV promoted NSCLC cell progression and glycolysis through miR-148a-3p/PDK4 axis. circFTO is a promising prognostic and diagnostic NSCLC biomarker and has the potential to be a candidate NSCLC therapy target.

**Supplementary Information:**

The online version contains supplementary material available at 10.1007/s00262-024-03634-4.

## Background

Lung cancer (LC) is among the most malignant cancers in the global, which has the highest mortality rate [1]. There are two classes of LC, small cell LC (SCLC) and non-small cell LC (NSCLC). NSCLC leads to 80–85% LC [2, 3]. In the past two decades, targeted therapies targeting driver gene positivity and immunotherapy targeting immune checkpoints have improved significantly survival of advanced NSCLC patients. However, drug resistance and long-term treatment outcomes remain unsatisfactory [4]. Therefore, there is an urgent demand to unravel further LC development mechanisms and to search for new biomarkers and therapeutic targets.

The tumor microenvironment (TME) is environment in which tumor cells live and is vital in cancer growth and metastasis [5]. Macrophages in TME are called tumor-associated macrophages (TAMs), which are an essential TME component. TAMs are closely associated with cancer cell proliferation (CP) and metastasis [6, 7]. TAMs include M1 macrophages and M2 macrophages (M2Ms), of which M1 macrophages exert a pro-inflammatory phenotype [8]. The M2Ms inhibit inflammation and promote tumor progression [9]. Nevertheless, the mechanisms by which M2Ms promote tumor progression are unclear.

TAMs regulate tumors progress by delivery bioinformatic molecules carried by their small extracellular vesicles (sEV) [10]. sEV are multivesicular bodies formed by intracellular lysosomal particle invaginations. They have a diameter of 30–150 nm (average 100 nm) and are present in almost all body fluids. EV contain cell components, such as DNA, lipids, RNA, metabolites, and cytoplasmic and surface proteins [11, 12]. As the studies on EV continue, it has been revealed that EV functions critically in intercellular communication (IC), participating in antigen presentation, cell growth, differentiation, migration, immune response to tumors, along with cancer cell invasion [13].

Circular RNAs (circRNAs) are non-coding RNAs (ncRNAs) made by selective splicing with a particular ring-like stable structure that is not degraded by RNA enzymes [14]. With recent developments in next-generation sequencing (NGS) techniques, differentially expressed circRNAs have been screened in distinguished cancers, unraveling the essential role of circRNA in cancer development [15, 16]. CircRNAs are now known to have multiple mechanisms, such like acting as miRNA sponges [17, 18], regulating variable splicing and transcription, translation, pseudogene generation, transport, and communication [19, 20]. In addition, due to their auxiliary role in translation, circRNAs can regulate gene expression [21]. Therefore, studying the function of circRNAs in cancer can help to find new therapeutic targets and provide new ideas for clinical treatment.

The current study investigated M2M-derived EV circRNA functions and mechanisms to develop NSCLC. We found that M2M-derived EV (M2-EV) promoted NSCLC cell proliferation, metastasis, and glycolysis. circFTO was significantly upregulated in M2-EV comparing to M0 macrophage-derived EV (M0-EV). In addition, circFTO expressed highly in NSCLC tumors and cells and negatively correlated to patient prognosis. Experiments confirmed that circFTO enhanced NSCLC proliferation and migration. Further studies revealed that circFTO targeted pyruvate dehydrogenase kinase 4 (PDK4)-mediated glycolysis through the sponge miR-148a-3p. Overall, our study has confirmed that circFTO from M2-EV promotes malignant progression and glycolysis in NSCLC cells via the miR-148a-3p/PDK4 axis.

## Methods

### Patient samples

Clinical samples were obtained from LC tissues and paracancerous tissues of 80 patients with NSCLC. The samples were extracted between 2016 and 2020 from patients in Department of Cardiothoracic Surgery, Xinhua Hospital, affiliated to Shanghai Jiao Tong University School of Medicine. Patients signed an informed consent before surgery. Ethics committee in Xinhua Hospital Affiliated to Shanghai Jiao Tong University supervised this investigation.

### Cell lines and cell culture

NSCLC cell lines (HCC1833, A549), normal human bronchial epithelial cells (BEAS-2B), and human monocytic leukemia cell line THP-1 (from the Shanghai Cell Bank, Chinese Academy of Sciences) used in the experiments were kept in the laboratory. We cultured the cells in RPMI-1640 medium supplemented with 10% FBS, 100 IU/mL penicillin, and 100 ug/mL streptomycin.

### Animal experiments

Experimental animals were healthy, 4–6-week-old male nude mice (BALB/c), 15 ~ 20 g. They were obtained from Shanghai Jihui Laboratory Animal Care Co., Ltd. Animal ethics committee in Xinhua Hospital approved the experiments.

### EV isolation and collection

After we induced the THP-1 cells into M2Ms, we washed the cells with phosphate-buffered saline (PBS) and added medium without FBS for 2 days. The culture medium of the M2Ms was harvested. We collected the EV from the culture medium by differential centrifugation as follows: medium centrifugation at 300 × g for 10 min; centrifugation of medium at 2 × g for 10 min to remove cells; centrifugation of medium at 10 × g for 0.5 h to remove cell debris; then ultracentrifugation at 120 × g for 90 min; resuspension of the EV in PBS; ultracentrifugation again at 120 × g for 90 min; finally, resuspension in PBS for collection into EP tubes. The EV’ size and concentration were quantified using a nanoparticle tracking analyzer (NTA).

### CCK8 cell viability test

Our team collected cells in logarithmic growth phase, which we inoculated into 96-well plates at 5000 cells/well. Then, 100 µL 10% CCK8 reagent was put into the corresponding wells every 12 h over the next 48 h. We incubated the cells at 37 ° away from light for 1 h. We detected OD values at wavelength λ = 450 nm by an end-point method using an enzyme labeler.

### Clone formation experiment

We collected cells in the logarithmic growth phase, which we inoculated into 6-well plates at 1000 cells/well. We placed them in a cell culture incubator for 2 weeks. They were subsequently fixed with 4% paraformaldehyde, stained with 0.1% Crystal Violet, washed with PBS, and analyzed using GraphPad.

### 5‑Ethynyl‑20‑deoxyuridine (EdU) assay

We collected cells in logarithmic growth phase and inoculated them into 24-well plates at 20,000 cells/well. Cells were placed in cell culture incubator for 1 days. Then, 0.5 mL EdU solution was put to every well, and we returned plate to cell culture incubator for 2 h for EdU labeling. The EdU solution was removed, and we washed the cells with PBS. Then, we added 250 μL 4% paraformaldehyde to every well for fixation. A glycine solution was put to neutralize paraformaldehyde, and 0.5% Triton X-100 was added to promote cell permeabilization. We finally stained cells using Hoechst 33,342. We photographed the EdU-treated cells utilizing inverted fluorescence microscope and counted the EdU-positive cell numbers.

### Wound healing assay

Our team collected cells in logarithmic growth phase and inoculated them into 6-well plates at 5 × 10^6^ cells/well. Cells were placed in a cell culture incubator for 1 days. Once the cells were fused to more than 95%, the original medium was discarded. The 6-well plate’s bottom was scratched perpendicularly utilizing 200-μL pipette tip. We washed non-adherent cells away with PBS. We took photographs under an inverted microscope with a 100 × field of view, and the change in the healing rate of the scratched area after 48 h was calculated.

### Transwell migration assay

We collected cells in the logarithmic growth phase to resuspend them in FBS-free medium at 2 × 10^5^ cells/mL. We inoculated a 200-µL cell suspension into the upper chamber and added 500 µL complete medium, including 15% FBS, to lower chamber. We cultured cells in a cell culture incubator for 1 days. Medium was discarded, and we fixed the cells in the lower chamber by adding 500 µL of 4% paraformaldehyde. The cells were then stained with 0.1% Crystalline Violet for 15 min, cleaned twice with PBS, and photographed using inverted microscope under a 100 × field of view. The stained cells were counted.

#### RNA extraction

We extracted total RNA utilizing TRIzol reagent following protocols. We synthesized complementary DNA (cDNA) employing a cDNA synthesis kit (Takara, Otsu, Japan). We performed RT-qPCR using a SYBR Green master mix (Vazyme, Nanjing, China). Relative gene expression was computed with 2-ΔΔCt approach. Primers employed in present investigation are listed in Table S1.

#### Western blotting

We inoculated different groups of cells into 6-well plates at 5 × 10^5^ cells/well. When cell fusion was 80% or more, the proteins were collected with 200 µL RIPA buffer (with 1:100 PMSF protease inhibitor and 1:100 phosphatase inhibitor) per well. Protein concentration was determined employing BCA protein concentration assay kit (Biyun Tian, Shanghai, China). We separated the proteins on SDS-PAGE gels and transferred proteins onto PVDF membranes. After blocking for 1 h with 5% skimmed milk powder, we washed membranes and incubated them overnight with primary antibodies. We then cleaned and incubated the membranes for 1 h with the corresponding secondary antibody. The ECL chemiluminescence detection solution (Vazyme, Nanjing, China) was added dropwise onto the PVDF membranes, and photographs were taken for imaging by exposing the membranes under an automated chemiluminescence image detection system (Tanon 5200). Image J was used for semi-quantitative analysis of the protein band gray values. Antibodies utilized in the current investigation included anti-CD63, anti-TSG101, anti-calnexin, anti-CD206, anti-PDK4, anti-LDHA, anti-HK2, anti-*β*-actin, and anti-*β*-tubulin.

#### Luciferase reporter assay

Our team inserted recombinant luciferase reporter plasmids at the potential miR-148a-3p binding site sequence in circFTO and PDK4 3'-UTR. Luciferase activity (LA) was captured utilizing a dual-LR assay system (Synergy LX; BioT, city, state, USA), which we normalized using Renilla luciferase.

#### Metabolic analysis

Cellular metabolic levels were investigated using a glucose uptake assay kit (Beyotime, Shanghai, China), lactate assay kit (Beyotime, Shanghai, China), and ATP content assay kit (Jiancheng Bioengineering Institute, Nanjing, China) following kits’ SOP.

#### Tumor xenograft formation

We injected viable HCC1833 or sh-circFTO-HCC1833 cells (2 × 10^6^) into the mouse's right flank to calculate tumor sizes each 5 days for 1 month utilizing Vernier caliper. Tumor volume was computed utilizing formula: length × width^2^ × 0.5. The relative expression of Ki67 was measured employing the IH method. Each group has 6 mice.

We stably transfected luminescence-labeled HCC1833 (Luc-HCC1833) cells with negative control (NC) for metastasis analyses. 2 × 10^5^ cells were injected into each nude mouse tail vein. After 4 weeks, our team validated lung metastasis by applying bioluminescence imaging system. The technician counted lung tissue metastatic foci after hematoxylin and eosin staining. Each group has 6 mice.

#### Statistical analyses

Statistician represented data by mean ± standard deviation (SD). Our team performed statistical analyses through GraphPad Prism (La Jolla, CA, USA) to define significances between groups. *P*-values ≤ 0.05 were considered as statistics significance. Our team employed 2-tailed Student’s t-tests to compute significances between 2 groups, while two-way ANOVA with post hoc Bonferroni tests or one-way ANOVA with Tukey tests were employed to define significant differences among >  = 3 groups.

## Results

### M2-EV promotes NSCLC CP and migration

**Fig. 1:**
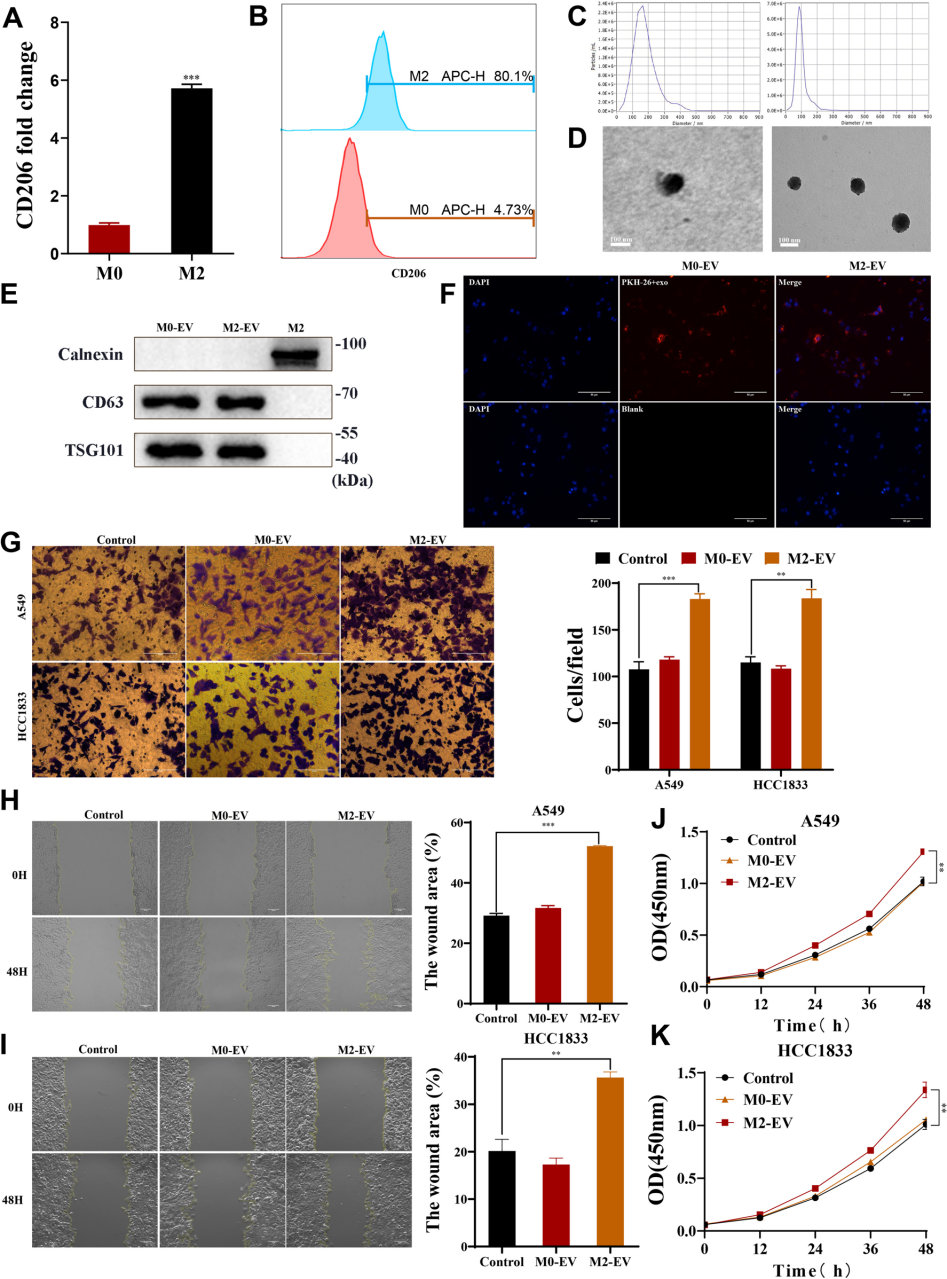
Identification of M2-EV and the promotional effect of M2-EV on NSCLC cells. **A**-**B** Expression of the M2M marker CD206 analyzed by RT-qPCR and flow cytometry. **C** The EV was examined by TEM. **D** Quantification of exosomes by nanoparticle tracking analysis. **E** The expression levels of CD63, TSG101, and calnexin were detected by western blot. **F** Fluorescence microscopy confirmed that the cells were able to take up M2-EV. **G** Transwell assays were used to study the migration ability of A549 and HCC1833 cells after treatment with M0-EV and M2-EV. Scale bar = 50 μm. **H**-**I** Wound healing assays in A549 and HCC1833 cells after treatment with M0-EV and M2-EV. Scale bar = 50 μm. **J**-**K** The viabilities of NSCLC cells were detected by CCK8 assays after treatment with M0-EV and M2-EV. Values are shown as the mean ± SD of three independent experiments. * *P* < 0.05; ** *P* < 0.01; *** *P* < 0.001

Accumulation studies have reported using human monocytic leukemia cell line THP-1 to research TAMs [22, 23]. We induced THP-1 cells into M0 and M2Ms and characterized them by RT-qPCR, western blot, and flow cytometry (Fig. 1A-B; Fig. S1A). Next, the cell culture medium (M2-CM) was collected after culturing the M2Ms in FBS-free medium for 24 h. The malignant behavior of the NSCLC cells post M2-CM and FBS-free medium treatment was examined by CCK8 assays (Fig. S1B-C), Transwell assays (Fig. S1D), and wound healing assays (Fig. S1E-F). The data showed that M2-CM significantly promoted A549 and HCC1833 cell proliferation and migration compared with the FBS-free medium group.

EV are known to be involved in IC within the TME. Therefore, we speculated whether M2-CM EV could promote NSCLC CP and metastasis. EV (M0-EV, M2-EV) was collected by ultracentrifugation from the M0-CM and M2-CM and identified (Fig. 1C-E). Next, we tested whether the NSCLC cells could take in the EV. EV were stained with PKH26 dye and added to the NSCLC cell medium for 1 days. Fluorescence microscopy confirmed that the NSCLC cells took in the PKH26-labeled EV (Fig. 1F). Transwell assays (Fig. 1G), wound healing assays (Fig. 1H-I), and CCK8 assays (Fig. 1J-K) showcased that M2-exo enhanced NSCLC cell proliferation and migration compared with the M0-exo treatment group.

### M2-EV promotes glycolysis in NSCLC

**Fig. 2:**
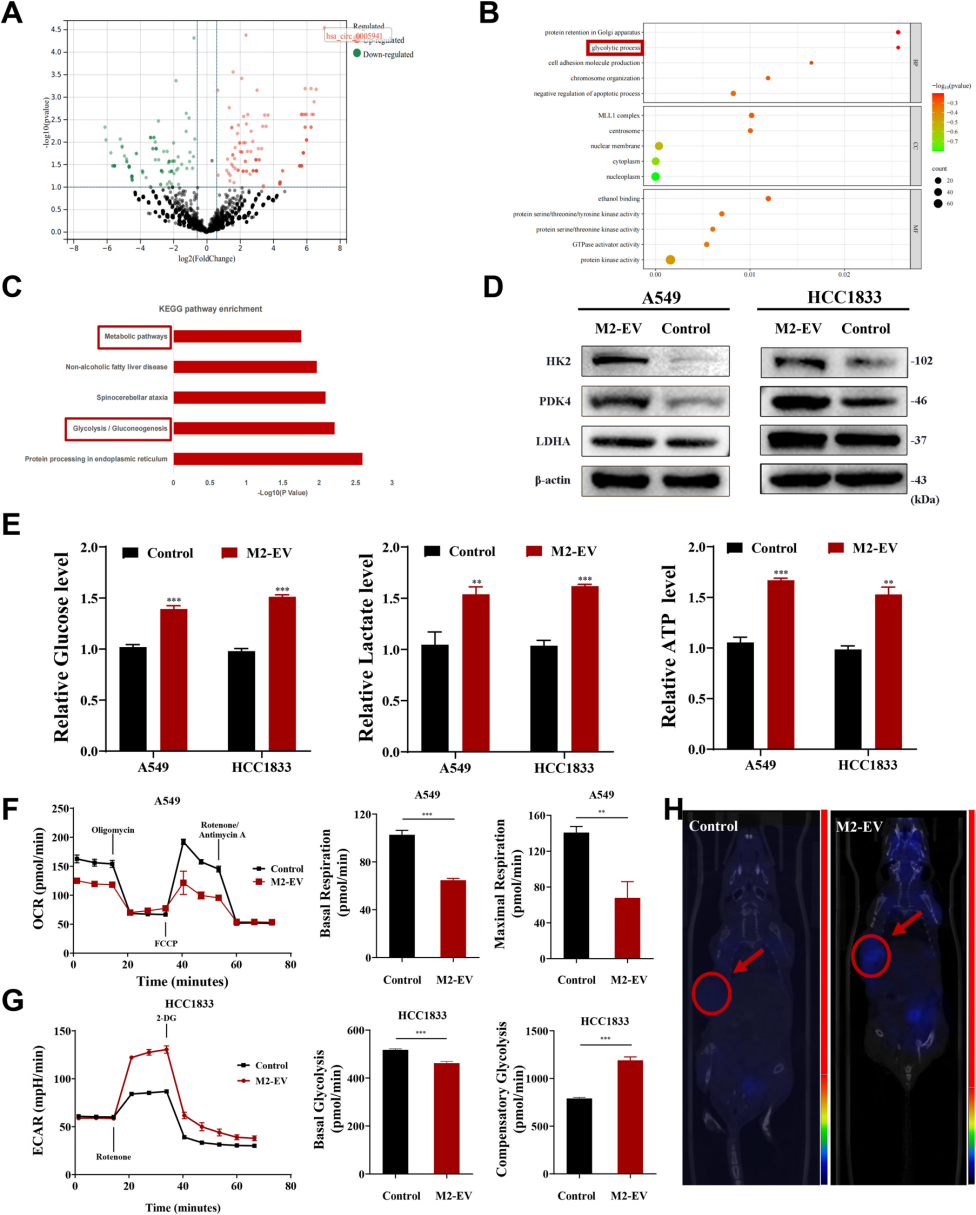
M2-EV promotes glycolysis in NSCLC. **A** Volcano plot of circRNA expression in M0-EV and M2-EV. **B**-**C** GO pathway enrichment analysis and KEGG pathway enrichment analysis were performed on the differentially expressed circRNAs. **D** The expression of glycolysis-related proteins in NSCLC cells was detected by western blotting. **E** Changes in relative glucose uptake, ATP levels, and lactate production after treatment with M2-EV. **F**-**G** Oxygen consumption rate (OCR) and extracellular acidification rate (ECAR) levels were measured using the Seahorse assays, and the basal/maximal respiration and glycolysis levels were calculated. **H** PET-CT was performed to assess glucose metabolism in mouse subcutaneous tumors. Values are shown as the mean ± SD of three independent experiments. * *P* < 0.05; ** *P* < 0.01; *** *P* < 0.001

NGS was used to detect circRNA expression in M0-EV and M2-EV. The data showcased that 299 circRNAs expressed differentially. Characterization showed that 142 and 157 circRNAs were up- and down-regulated, respectively (Fig. 2A). KEGG and GO pathway enrichment analyses were made utilizing the DAVID database (Fig. 2B-C). Among the enriched pathways, glycolysis-related pathways were found to promote tumor progression. Therefore, we speculated whether M2-EV could promote glycolytic progression. Western blotting confirmed that M2-EV could promote the expression of the glycolysis-related proteins HK2, LDHA, and PDK4 in NSCLC cells (Fig. 2D). Meanwhile, we found that cellular glucose uptake, ATP levels, and lactate production increased after treatment with M2-EV (Fig. 2E). Next, the Seahorse assay was employed to verify the M2-EV effects upon glycolysis in the NSCLC cells. The outputs showed that the cellular respiration level decreased and the glycolysis level increased after treatment with M2-EV (Fig. 2F-G and Fig. S2A-B). PET-CT scanning indicated that the M2-EV treatment could significantly enhance glucose metabolism in subcutaneous tumors in mice (Fig. 2H). The above data indicated that M2-EV promoted the glycolytic process in NSCLC cells.

### M2-EV enhances NSCLC cell proliferation and migration through circFTO

**Fig. 3:**
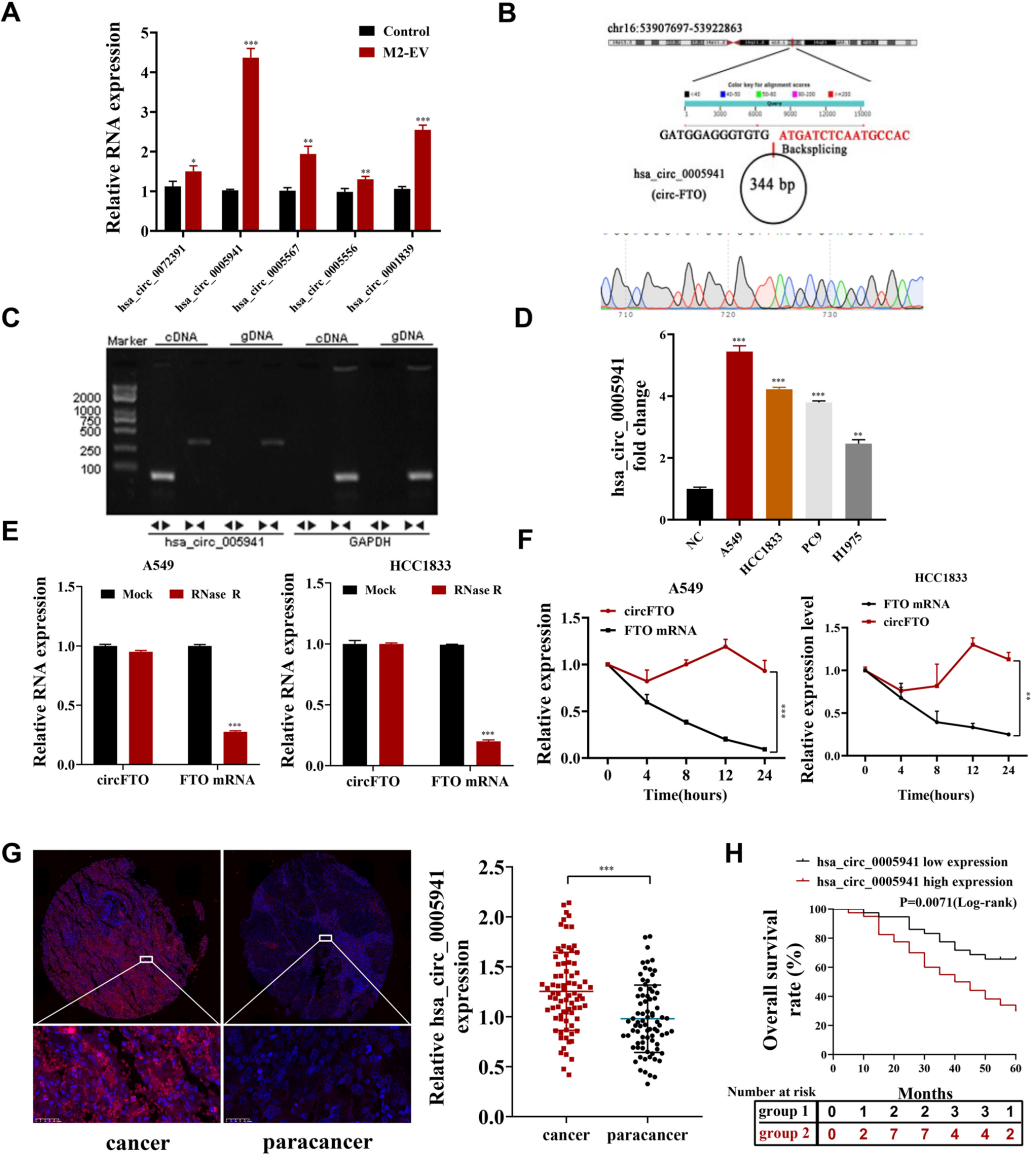
M2-EV delivers circFTO into NSCLC cells. **A** The expression of five circRNAs in A549 cells after treatment with M2-EV was detected by RT-qPCR. **B** The back-splice junction site of circFTO was verified by Sanger sequencing. **C** circFTO was detected by qRT–PCR and agarose gel electrophoresis using divergent and convergent primers. **D** The expression of circFTO in NSCLC cells was analyzed by RT-qPCR. **E** The expression of circFTO and FTO mRNA in NSLCL cells after treatment with RNase R. **F** RNA abundance of circFTO and FTO after treatment with actinomycin D. **G** circFTO expression levels in NSCLC cancer and paracancer was verified by FISH. **H** Kaplan–Meier analysis showed that expression of circFTO was associated with overall survival in NSCLC patients. Values are shown as the mean ± SD of three independent experiments. * *P* < 0.05; ** *P* < 0.01; *** *P* < 0.001

Five upregulated circRNAs in NSCLC cells with or without M2-EV treatment were selected and verified by RT-qPCR (Fig. 3A). The outputs showed that has_circ_0005941 expression was significantly increased. Hsa_circ_0005941 was generated by the FTO gene located on chr16:53,907,697–53922863. The circFTO back-splice site was verified by Sanger sequencing (Fig. 3B). Divergent primers for circFTO could be amplified via PCR from cDNA yet not gDNA (Fig. 3C). Next, we examined circFTO expression in NSCLC cell lines to find that it expressed highly in NSCLC cells. The highest expression was in A549 and HCC1833 cells (Fig. 3D). Therefore, both A549 and HCC1833 cells were selected for the following studies. Compared with linear FTO mRNA, circFTO was more stable post RNase R and actinomycin D treatments (Fig. 3E-F).

CircFTO expression in NSCLC tissues was verified through FISH. Outputs showcased that circFTO expression incremented in LC tissues (80 cases) comparing to the adjacent normal tissues (Fig. 3G). circFTO expression level also correlated to the tumor’s pathological grade and TNM stage (Table S2). Kaplan–Meier analysis of correlations between circFTO expression and overall survival revealed that patients having a high circFTO expression had a poor prognosis (Fig. 3H).

To confirm whether M2-exo regulates NSCLC progression via circFTO, we established a stable circFTO-knockdown THP-1 cell line by lentiviral transfection. Transfection efficiency was verified through RT-qPCR (Fig. S3A). The circFTO-knockdown THP-1 cells were induced to the M2 type, and their secreted EV were collected. Sh-circFTO-M2-exo and M2-exo were added to A549 and HCC1833 cell media, respectively. CCK8 assays and Transwell assays (Fig. S3B-C) showed that downregulating circFTO decreased the promotion effect of circFTO on NSCLC cell malignant progressions, including migration and proliferation. The above results demonstrated that M2-exo enhanced NSCLC cell proliferation and migration via the circFTO delivery.

### Knockdown of circFTO expression suppresses NSCLC cell proliferation and migration

**Fig. 4:**
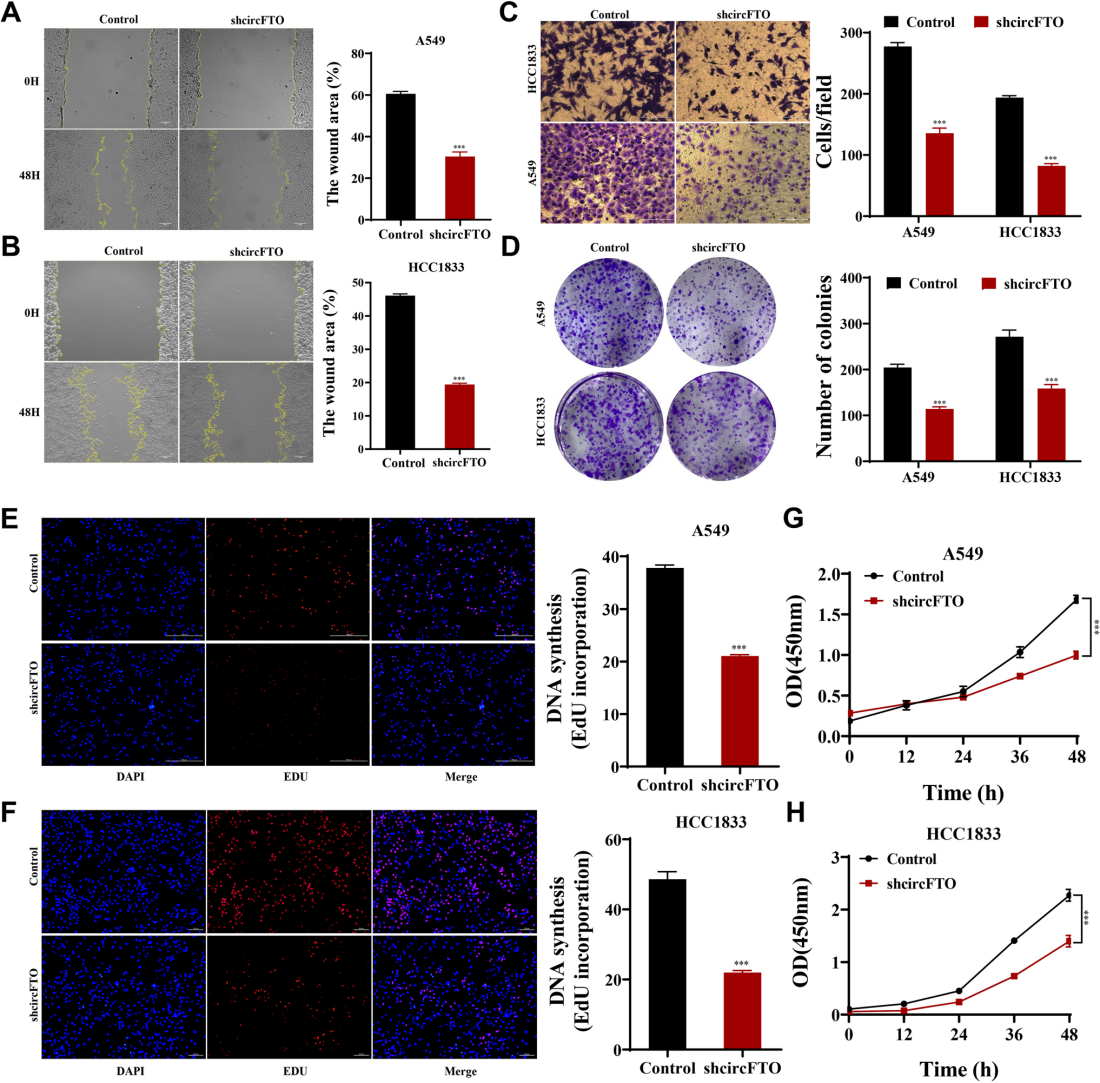
circFTO facilitates the proliferation and migration of NSCLC cells in vitro. **A**-**B** Knockdown of circFTO expression significantly inhibited cell migration as determined by wound healing assays. **C** Knockdown of circFTO expression significantly inhibited cell migration as determined by Transwell assays. **D**-**H** Cell proliferation was analyzed by CCK8 assays, colony formation, and EdU assays. Knockdown of circFTO expression significantly inhibited cell proliferation in A549 and HCC1833 cells. Values are shown as the mean ± SD of three independent experiments. * *P* < 0.05; ** *P* < 0.01; *** *P* < 0.001

Our team established stable circFTO-knockdown cell lines by lentiviral transfection and verified the effectiveness of circFTO-knockdown by RT-qPCR (Fig. S4A). Wound healing and Transwell assays showed that circFTO-knockdown significantly suppressed NSCLC cell migration ability (Fig. 4A-C). Meanwhile, CP was assessed by clone formation assays (Fig. 4D), EdU assays (Fig. 4E-F), and CCK8 assays (Fig. 4G-H). The data showed that circFTO-knockdown inhibited CP significantly. The findings unraveled that knocking down circFTO expression inhibited NSCLC cell proliferation and migration.

### Knockdown of circFTO expression suppresses NSCLC cell growth

**Fig. 5:**
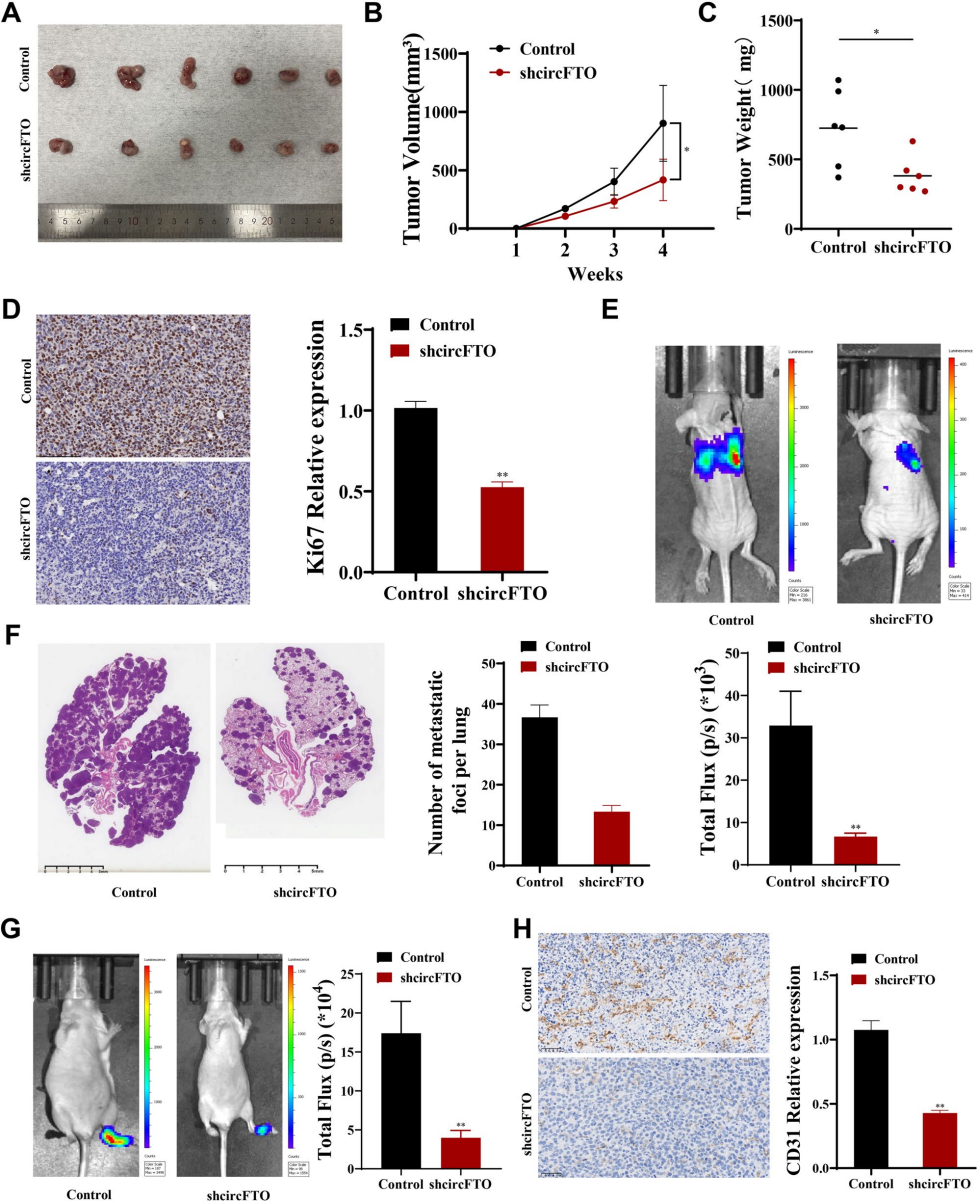
Knockdown of circFTO expression inhibits the growth and metastasis of NSCLC cells in vivo. **A** Representative picture of mouse subcutaneous tumors (*n* = 6 for each group). **B**-**C** Curves of tumor volumes and weights in circFTO-knockdown and control groups. **D** The expression of Ki-67 in mouse subcutaneous tumors was detected by IHC staining. **E** Representative images and analysis of luminescence intensity in tail vein tumor metastasis mouse models (*n* = 3 for each group). **F** HE-stained images of metastatic nodules in the lungs of mice and statistics on the number of nodules. **G** Representative picture and analysis of luminescence intensity in plantar lymphatic transfer models (*n* = 3 for each group). **H** The expression of CD31 in mouse plantar lymphatic transfer tumors was detected by IHC staining. Values are shown as the mean ± SD of three independent experiments. * *P* < 0.05; ** *P* < 0.01; *** *P* < 0.001

To evaluate circFTO effects on NSCLC cells in vivo, we injected circFTO-knockdown HCC1833 cells and normal HCC1833 cells subcutaneously into BALB/cA nude mice. In this subcutaneous tumor formation model, circFTO-knockdown suppressed tumor growth in volume and weight compared to control group (Fig. 5A-C). Simultaneous IHC staining showcased that Ki-67 expression level within tumors was decreased in circFTO-knockdown group (Fig. 5D). In a caudal vein transfer model, bioluminescence imaging showed a significantly lower fluorescence intensity and ratio in lungs of circFTO-knockdown group than control group (Fig. 5E). Meanwhile, HE staining demonstrated that circFTO knockdown significantly reduced the metastatic nodule numbers in lungs (Fig. 5F). In studies of the plantar lymph node metastasis model, bioluminescence imaging showed that circFTO knockdown significantly inhibited lymph node metastasis of the plantar tumors (Fig. 5G), and IHC staining showed that CD31 expression decreased (Fig. 5H). The above results suggested that circFTO knockdown inhibited NSCLC proliferation and metastasis.

### circFTO functions as ceRNA for miR-148a-3p to regulate cancer progression

**Fig. 6:**
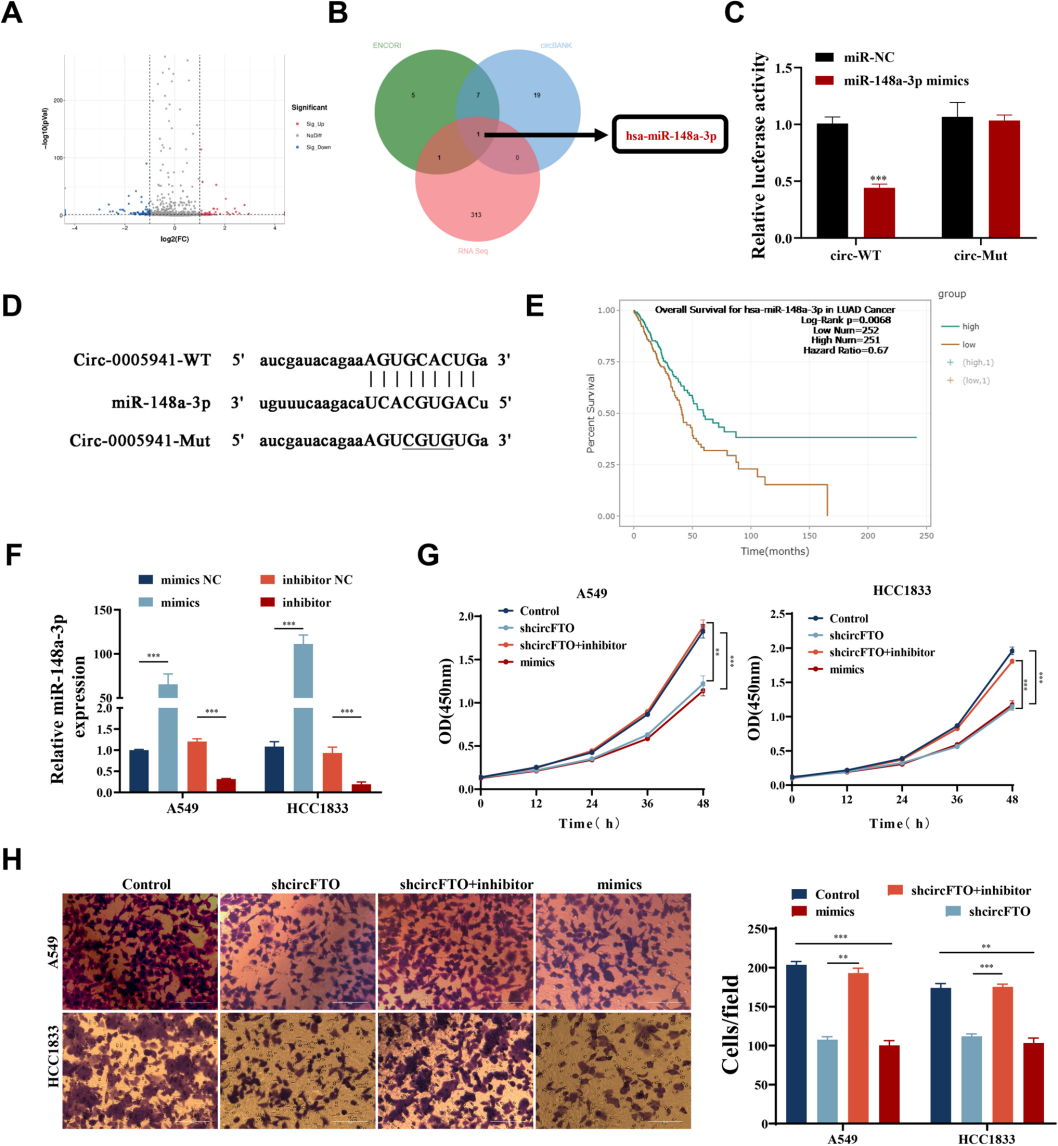
miR-148a-3p is a target of circFTO. **A** Volcano plot of miRNA expression in circFTO-knockdown cells analyzed by RNA-seq. **B** Venn diagram showing the overlap of the target miRNAs of circFTO predicted by circBANK, ENCORI, and the results of RNA-seq. **C**-**D** An LR assay was used to confirm the targeting relationship between circFTO and miR-148a-3p. **E** ENCORI database showed the relationship between miR-148a-3p expression and patient prognosis. **F** Transfection efficiency of miR-148a-3p mimics and miR-148a-3p inhibitor verified by RT-qPCR. **G** CCK8 assays of NSCLC cells were performed to evaluate CP. **H** Transwell assays were used to investigate the migratory abilities of NSCLC cells. Values are shown as the mean ± SD of three independent experiments. * *P* < 0.05; ** *P* < 0.01; *** *P* < 0.001

We hypothesized that circFTO might function as miRNA sponge in NSCLC. miRNA sequencing showed that circFTO-knockdown resulted in different expressions of miRNA in HCC1833 cells (Fig. 6A). KEGG pathway enrichment analyses similarly showcased that metabolic pathways were affected significantly (Fig. S4B). To identify the downstream miRNAs of circFTO, RNA-seq was performed on the circFTO-knockdown HCC1833 cells and cross-analyzed with the predicted results from two miRNA target prediction databases (circBANK, ENCORI). The output data showcased that miR-148a-3p was the only circFTO downstream target (Fig. 6B). Targeting correlation between miR-148a-3p and circFTO was verified through LR assays (Fig. 6C-D). The outputs showed that miR-148a-3p mimics significantly inhibited circFTO-WT LA, though LA of circFTO-MUT did not alter significantly. The ENCORI database showed that patients having higher miR-148a-3p expression levels have longer survival times (Fig. 6E). To validate the miR-148a-3p effects upon circFTO expression, HCC1833 and A549 cells were transfected with mimics and miR-148a-3p inhibitor, respectively. Transfection efficiency was verified by RT-qPCR (Fig. 6F). We performed salvage assays to further research miR-148a-3p and circFTO functions during NSCLC progression. CCK8 assay results showed that the inhibitory effect caused by circFTO knockdown could be reversed post miR-148a-3p inhibitor transfections (Fig. 6G). Similarly, the Transwell assays illustrated that miR-148a-3p inhibitors could reverse NCSLC cell migration inhibition ability via circFTO knockdown (Fig. 6H). The data revealed that miR-148a-3p functioned critically in circFTO-mediated regulation effect on the progress of NSCLC.

### circFTO regulates PDK4 expression through miR-148a-3p

**Fig. 7:**
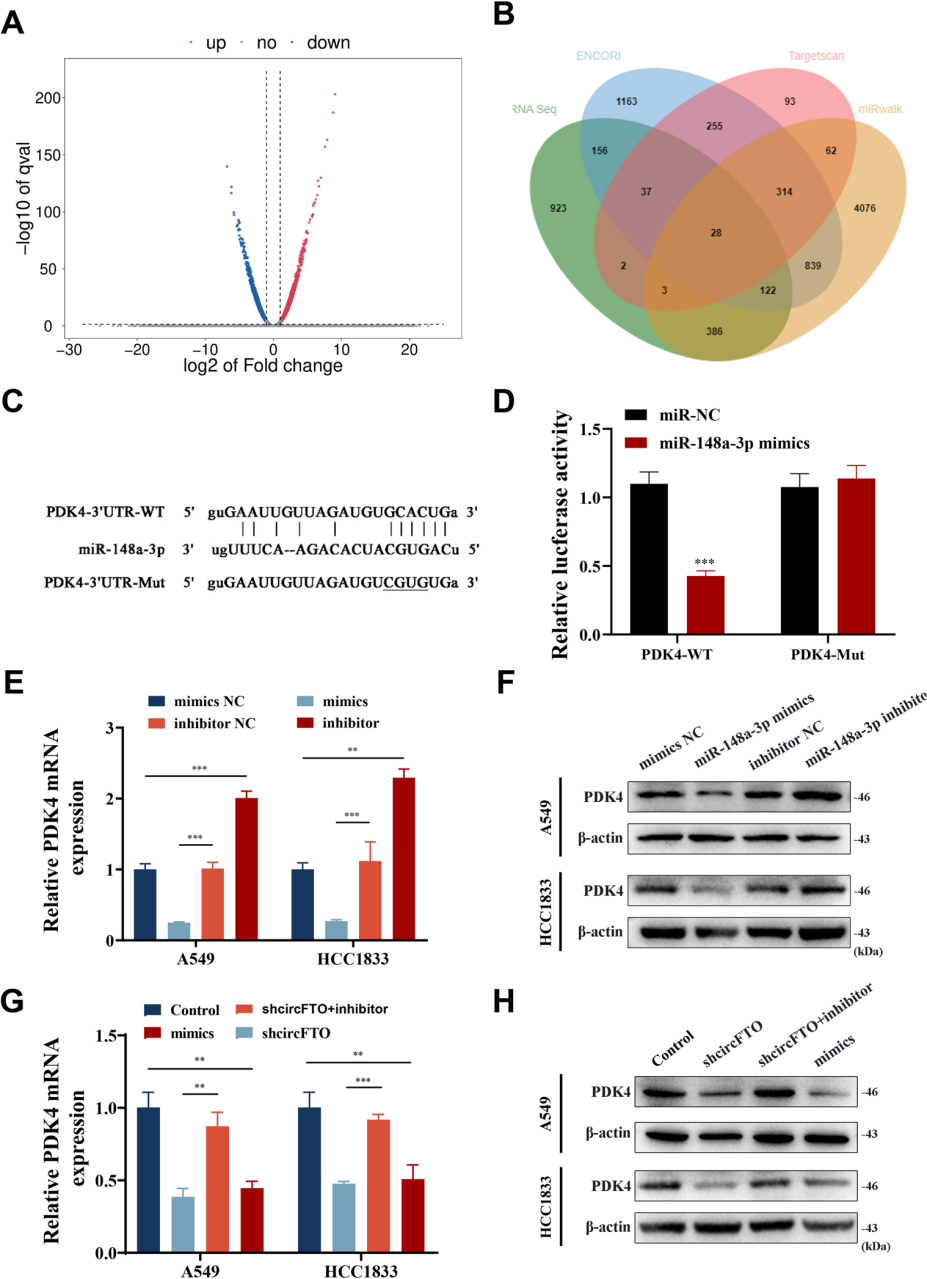
circFTO regulates PDK4 expression through miR-148a-3p. **A** Volcano plot of mRNA expression in circFTO-knockdown cells analyzed by RNA-seq. **B** Venn diagram showing the overlap of the target mRNAs of miR-148a-3p predicted by Targetscan, miRwalk, ENCORI, and the results of RNA-seq. **C**-**D** An luciferase report analysis assay was used to confirm the targeting relationship between miR-148a-3p and PDK4. **E**-**H** The expression levels of PDK4 mRNA **E** and protein **F** of NSCLC cells transfected with miR-148a-3p mimics and miR-148a-3p inhibitor. **G**-**H** The expression levels of PDK4 mRNA **G** and protein **H** were significantly downregulated in circFTO-knockdown NSCLC cells, and these effects could be reversed by transfection of the miR-148a-3p inhibitor. Values are shown as the mean ± SD of three independent experiments. *P < 0.05; **P < 0.01; ***P < 0.001

To investigate molecular mechanism of miR-148a-3p in NSCLC, miR-148a-3p downstream targets were analyzed utilizing RNA-seq combined with bioinformatics analysis results from Targetscan, ENCORI, and miRwalk, which showed 28 possible genes targeted through miR-148a-3p (Fig. 7A-B). The glycolysis-related protein PDK4 was found among the 28 proteins, so we selected PDK4 for downstream analysis. Next, mutant and wild-type luciferase report analysis plasmids for the PDK4 3'-UTR were made. Data showcased that miR-148a-3p mimic transfections significantly suppressed PDK4-WT (wild) fluorescence intensity, whereas there was no significant alteration in PDK4-MUT (mutant) luciferase activities (Fig. 7C-D). RT-qPCR and western blot data demonstrated that PDK4 was regulated negatively by miR-148a-3p (Fig. 7E-F). In addition, circFTO-knockdown (shcircFTO) similarly inhibited mRNA and protein levels with regard to PDK4, and miR-148a-3p inhibitor ultimately rescued this inhibition (Fig. 7G-H). In conclusion, our results confirmed that circFTO upregulated PDK4 expression through the sponge miR-148a-3p.

### M2-EV promotes glycolysis and NSCLC progression through circFTO/miR-148a-3p/PDK4 axis

**Fig. 8:**
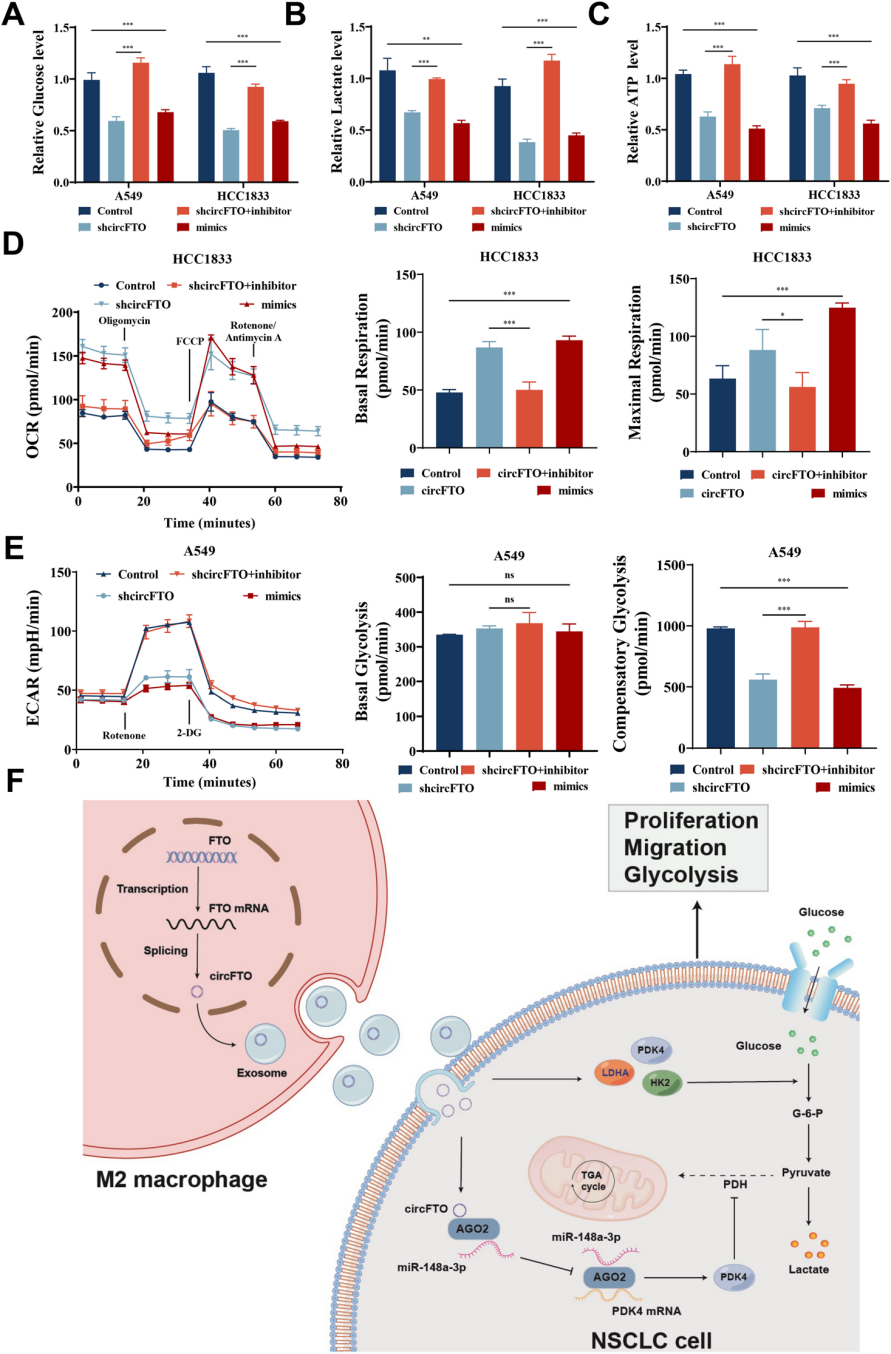
Detection of glycolysis-related indicators. **A**-**C** Relative glucose uptake, ATP levels, and lactate production in NSCLC cells transfected with miR-148a-3p mimics/inhibitor treatment and circFTO-knockdown. **D**-**E** OCR and ECAR levels were measured in NSCLC cells transfected with miR-148a-3p mimics/inhibitor treatment and circFTO-knockdown. **F** Schematic illustration of M2-EV delivering circFTO to promote NSCLC cell malignant progression and glycolysis. Values are shown as the mean ± SD of three independent experiments. * *P* < 0.05; ** *P* < 0.01; *** *P* < 0.001

PDK4 positively regulates glycolysis in some tissues and cancers. To research whether circFTO could promote NSCLC cell glycolysis and progression through miR-148a-3p/PDK4 axis, we performed a Seahorse assay and relevant functional tests relating to glucose metabolism. The result found that circFTO-knockdown suppressed glucose uptake, ATP levels, and lactate production in NSCLC cells. Also, a miR-148a-3p suppressor was able to reverse this process, while miR-148a-3p mimic inhibited glucose uptake, lactate, and ATP production in NSCLC cells (Fig. 8A-C). Meanwhile, Seahorse assay data showed that circFTO knockdown or miR-148a-3p overexpression increased cellular respiration levels and decreased glycolysis levels. The miR-148a-3p inhibitor could reverse circFTO-knockdown effects completely (Fig. 8D-E). These results demonstrate that circFTO could promote NSCLC cell glycolysis and progression through the miR-148a-3p/PDK4 axis.

Similarly, our team found that glycolysis, ATP levels, and lactate production in NSCLC were attenuated by sh-circFTO-M2-EV compared to M2-EV treatment (Fig. S5A-C). The Seahorse assay also showed the same results (Fig. S5D-E). The above results suggest that M2-exo promoted NSCLC glycolysis and progression via the circ-FTO/miR-148a-3p/PDK4 axis.

## Discussion

The regulatory role of the TME on tumors has been discussed [24], for which the tumor-promoting role of M2Ms has been increasingly reported [10, 22]. Recently, it has been shown that M2Ms regulate cancer progression through their secreted EV [10]. Therefore, we wanted to understand whether M2Ms could go through the exosomal pathway to regulate lung carcinogenesis and development. The current investigation unraveled that M2-CM promoted NSCLC cell proliferation and migration. We further verified that part of promotion effect was achieved through M2-EV. Meanwhile, we found that M2-exo promoted aerobic glycolysis in LC cells.

EV are found in almost all body fluids and contain many components of cells, including DNA, RNA, and proteins [25]. With the in-depth studies on EV, it has been found that EV function essentially in ICs, which participate in antigen presentation, growth, immune response to tumors, etc. [13]. For example, Lan and colleagues discovered that M2M EV enhance colon cancer cell proliferation and invasion by delivery miRNA [10], and Yang and colleagues found that miR-423-5p promotes gastric cancer cell proliferation and metastasis through suppressing SUFU protein expressions [26]. These roles of EV give them great potential as cancer therapeutic biomarkers.

With the advancement of technology, more than 100,000 circRNAs have been identified [27]. There is growing evidence that circRNAs function essentially in LC development and influence cellular functions [20]. They can regulate CP [28], migration, and apoptosis [29], induce multidrug resistance (MDR), and regulate the TME through different signaling pathways [30]. circRNAs have several functions in TME, promoting or inhibiting immune system and angiogenesis, enhancing endothelial cell permeability, promoting tumor metastasis, causing ECM remodeling, and supporting tumor progression [31, 32]. Therefore, we performed RNA-seq on circRNAs in M2-EV and found that circFTO expression was elevated in M2-EV. Further studies on circFTO have revealed that circFTO expression is upregulated significantly in LC tissues and cells comparing to paracancerous tissues and normal bronchial epithelial cells. Patients having high circFTO expression have a relatively poor prognosis. Inhibition of circFTO expression significantly reduced the malignancy of NSCLC cells, confirming that circFTO functions to promote NSCLC malignant progression by M2-EV.

More investigations have reported that circRNAs act as miRNA sponges to regulate miRNAs and downstream target gene expressions [33, 34]. Our team validated miR-148a-3p as a circFTO downstream target by RNA-seq, informatics prediction, and luciferase report analysis validation. Cellular experiments verified that a miR-148a-3p suppressor could rescue inhibitory effect by interfering with circFTO in NSCLC cell proliferation and migration. Our data advise that circFTO regulates NSCLC proliferation and migration via sponge miR-148a-3p, which could inhibit tumorigenesis development. Xu et al. reported that miR-148a-3p suppresses bladder cancer proliferation and migration via regulating Roc-1 expression [35]. Zeng et al. found that circANKS1B regulates breast cancer by promoting the transcription factor UF1 expression via the sponge miR-148a-3p [36]. To elucidate molecular mechanism of miR-148a-3p in LC progression, our team combined RNA-seq and informatics prediction results to identify PDK4 as a possible miR-148a-3p downstream target. We confirmed the relationship by dual-LR assay validation and RT-qPCR.

Otto Warburg found that cancer cells could metabolize glucose by glycolysis even in an adequate oxygen supply. Since then, aerobic glycolysis has been recognized as an essential cancer hallmark, providing cancer cells with an advantage in bioenergetics, biosynthesis, and redox homeostasis [37]. Pyruvate dehydrogenase complex (PDC) catalyzes pyruvate to acetyl coenzyme A in mitochondria and is a crucial regulator of glucose oxidation. PDK4 is a key enzyme regulating PDC activity, which is a crucial pyruvate oxidation and glucose maintenance homeostasis regulator in vivo, promoting the Warburg effect and tumor growth. Li et al. reported that miR-182/PDK4 axis regulates lung tumorigenesis through pyruvate dehydrogenase and adipogenesis regulations [38]. Xu and colleagues revealed that lncRNA PCAT1 enhances proliferation and glycolysis of laryngeal cancer cells through miR-182/PDK4 axis [39]. Our findings suggested that circFTO regulated tumor progression and aerobic glycolysis through the miR-148a-3p/PDK4 axis.

To conclude, the cumulative data suggest that M2Ms promote progression and glycolysis in NSCLC via EV-circFTO delivery. The circFTO effects on NSCLC progression and glycolysis are mediated through the miR-148a-3p/PDK4 axis. Therefore, M2M-EV-derived circFTO promotes NSCLC progression and glycolysis via the miR-148a-3p/PDK4 axis (Fig. 8F).

### Supplementary Information

Below is the link to the electronic supplementary material.

Supplementary file1 (PDF 2158 KB)

Supplementary file2 (DOCX 2512 KB)

## Data Availability

The datasets used and/or analyzed during the current study are available from the corresponding author on reasonable request.
