# Supplementary material for: circFTO from M2 macrophage-derived small extracellular vesicles (sEV) enhances NSCLC malignancy by regulation miR-148a-3pPDK4 axis
Source: Cancer Immunol Immunother. 2024 Mar 30;73(5):91. doi: 10.1007/s00262-024-03634-4 (PMC10981622; doi:10.1007/s00262-024-03634-4)
Supplement: Supplementary file 1 — Supplementary file1 (PDF 2158 KB) [file 262_2024_3634_MOESM1_ESM.pdf]

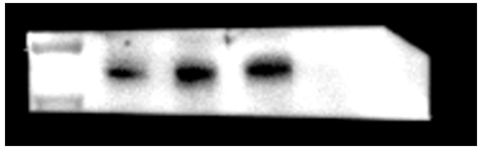

CD206

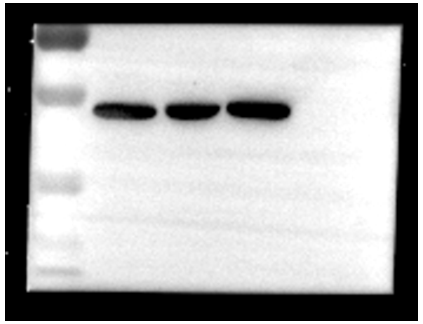

B-tubulin

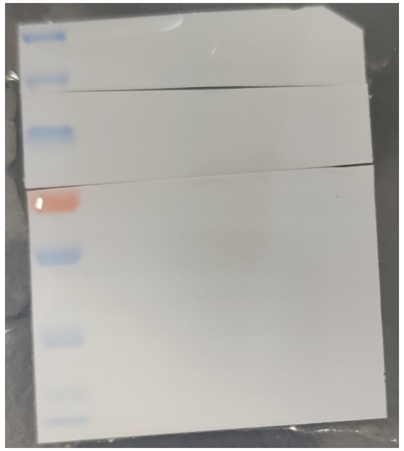

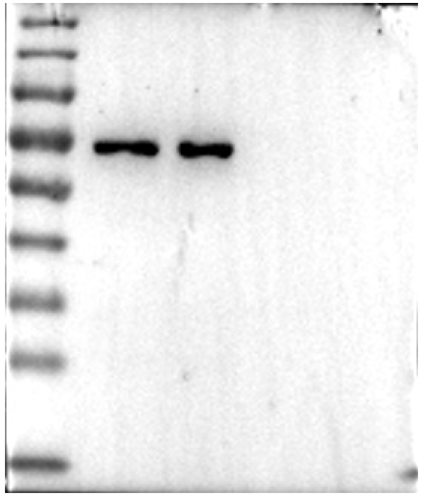

CD63

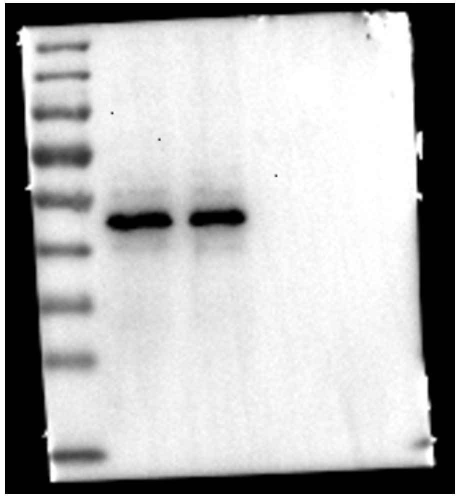

TSG101

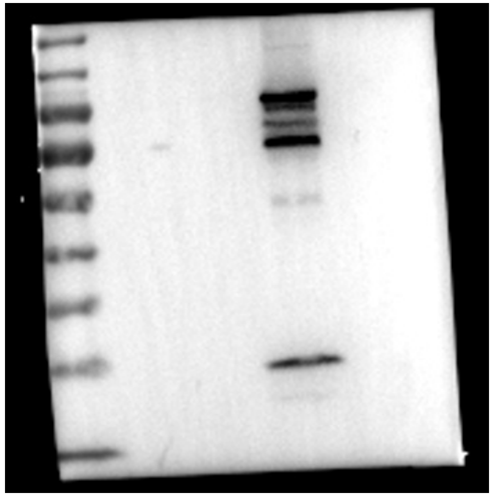

CALNEXIN

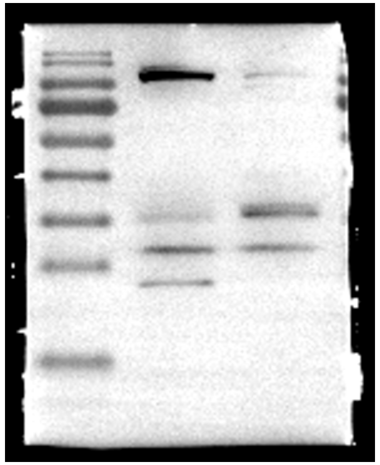

HK2

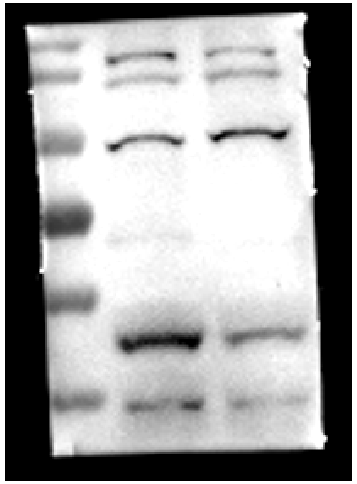

PDK4

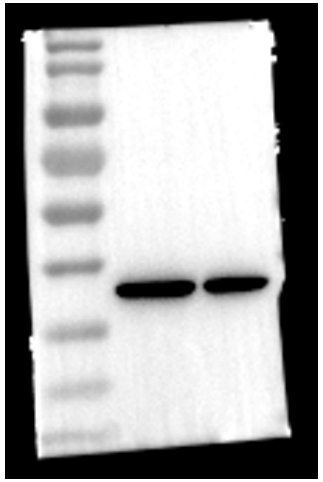

LDHA

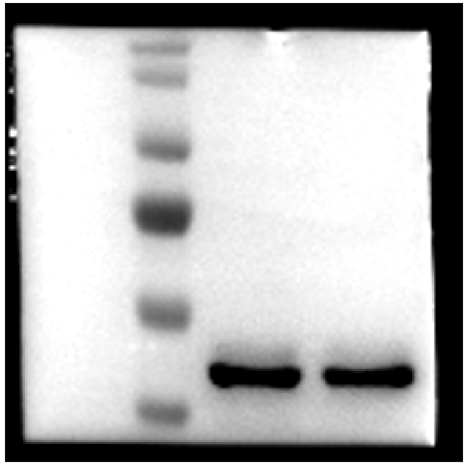

β-actin

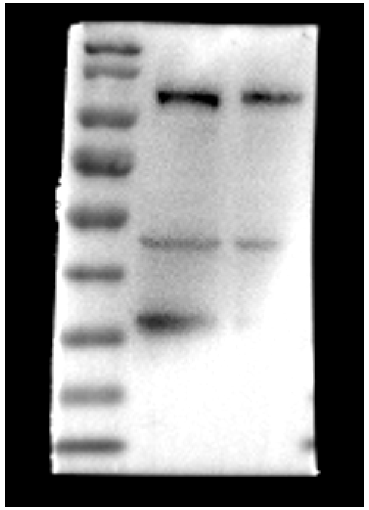

HK2

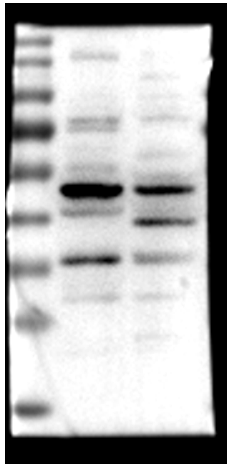

PDK4

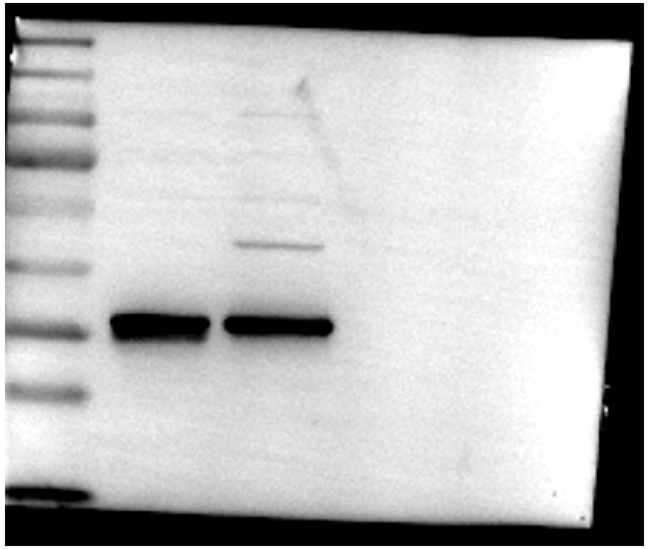

LDHA

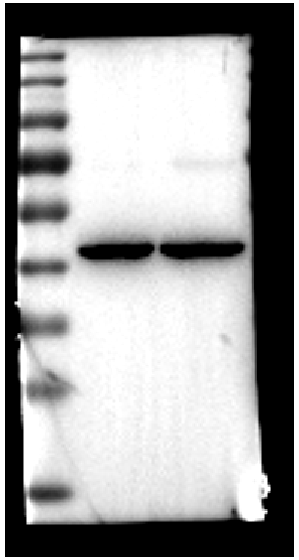

β-actin

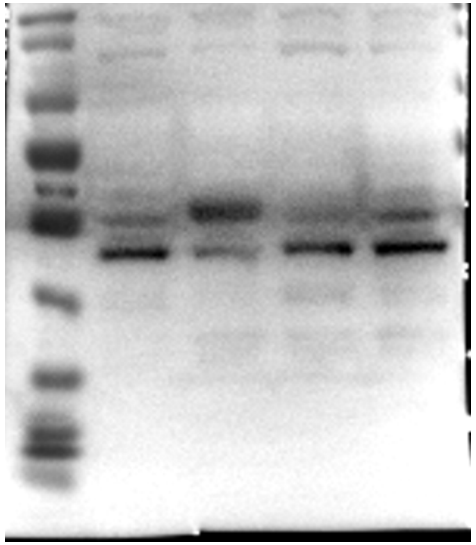

PDK4

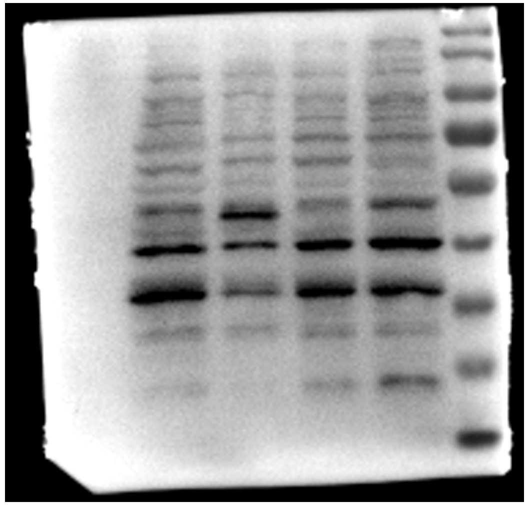

PDK4

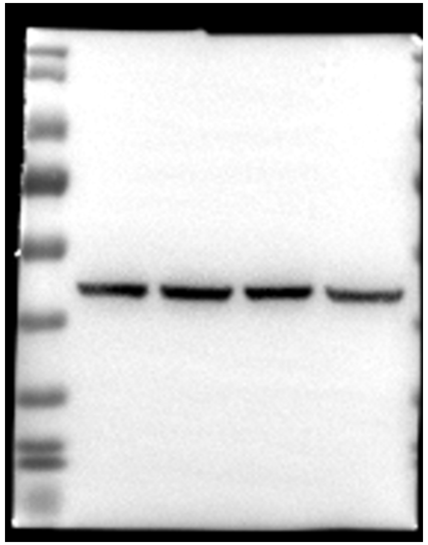

β-actin

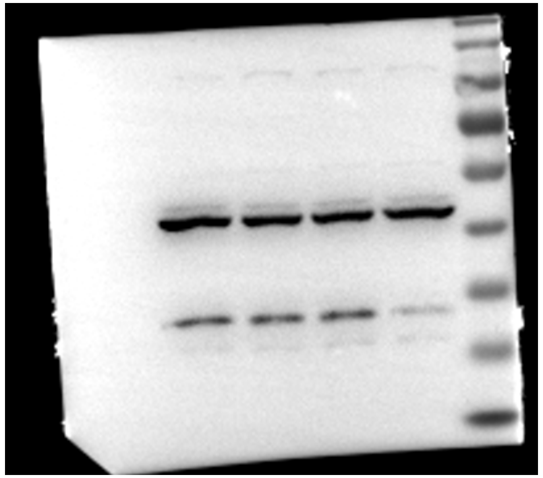

β-actin

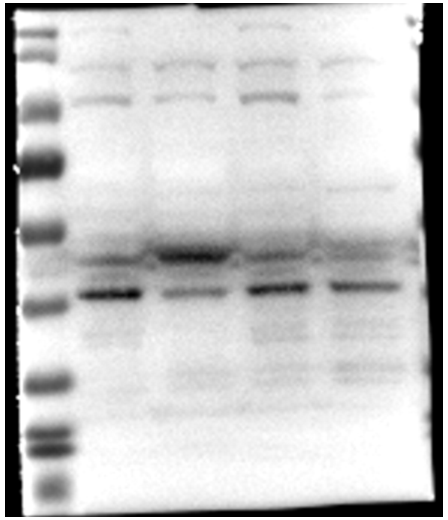

PDK4

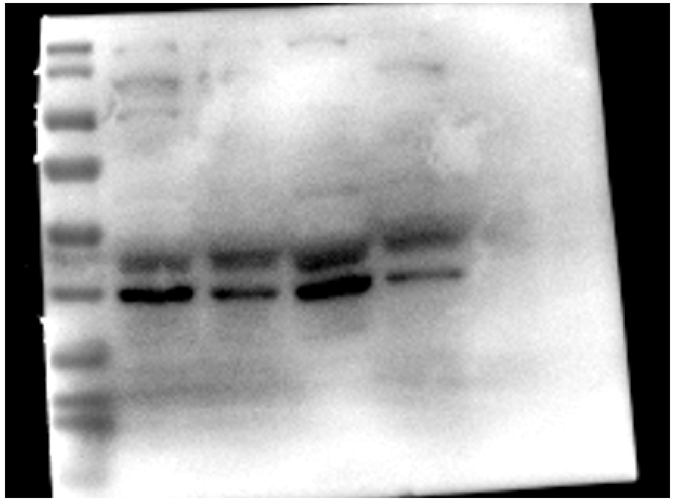

PDK4

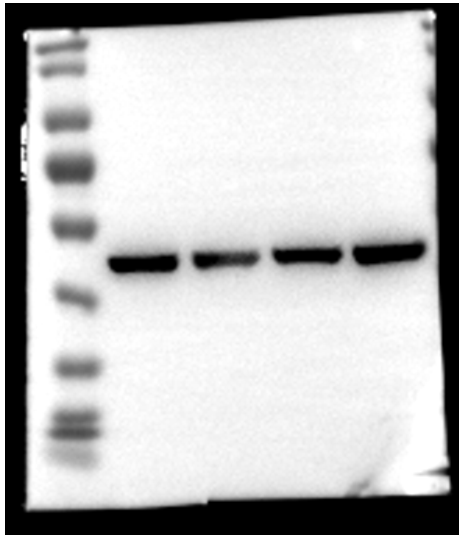

β-actin

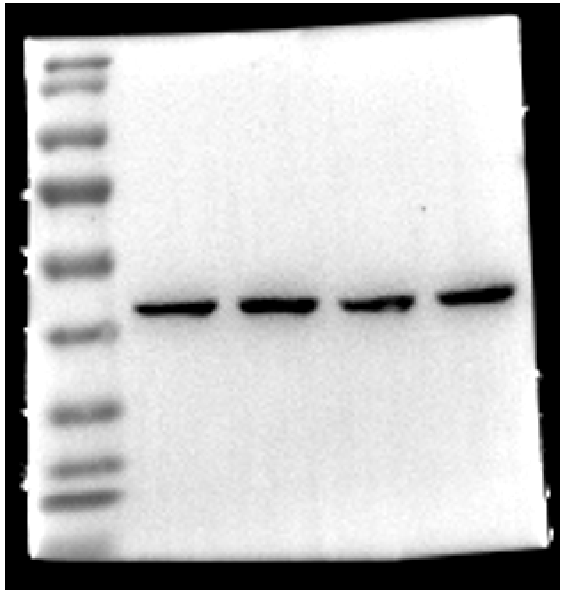

β-actin
