# Supplementary material for: circFTO from M2 macrophage-derived small extracellular vesicles (sEV) enhances NSCLC malignancy by regulation miR-148a-3pPDK4 axis
Source: Cancer Immunol Immunother. 2024 Mar 30;73(5):91. doi: 10.1007/s00262-024-03634-4 (PMC10981622; doi:10.1007/s00262-024-03634-4)
Supplement: Supplementary file 2 — Supplementary file2 (DOCX 2512 KB) [file 262_2024_3634_MOESM2_ESM.docx]

**Supplementary material**

Figure S1.M2-CM promotes the proliferation and migration of NSCLC cells. (A) The expression levels of CD206 were detected by western blot. (B-C) The viabilities of the NSCLC cells were detected by CCK8 assays after treatment with M2-CM. (D-F) The migration abilities of the NSCLC cells were detected by Transwell and wound healing assays after treatment with M2-CM. Scale bar = 50 μm. Values are shown as the mean ± SD of three independent experiments. * P < 0.05; ** P < 0.01; *** P < 0.001.


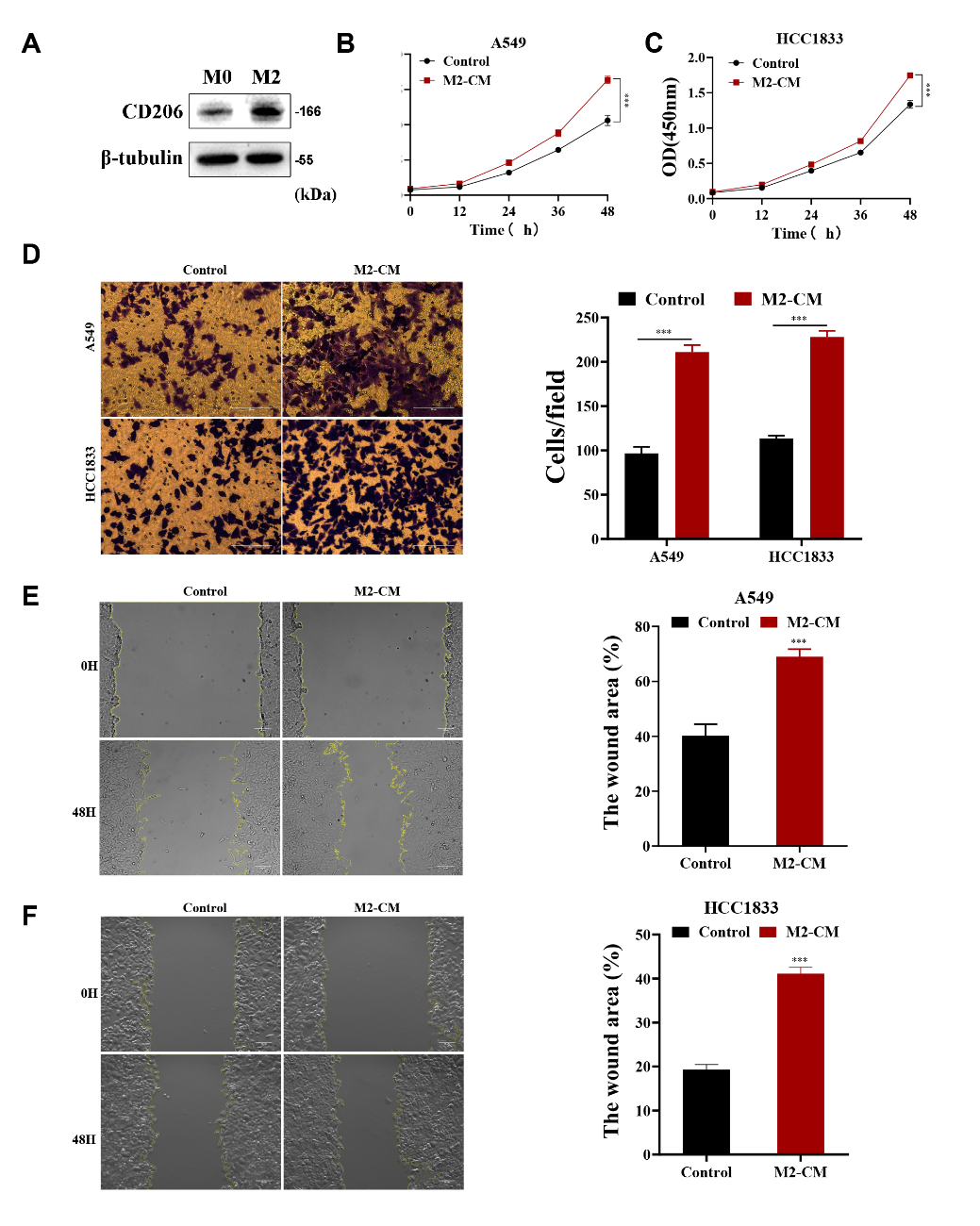


Figure S2.OCR and ECAR in NSCLC cells after treatment with M2-CM. (A) Changes in OCR and the basal/maximal respiration of HCC1833 cells after treatment with M2-CM. (B) Changes in ECAR and glycolysis levels of A549 cells after treatment with M2-CM.


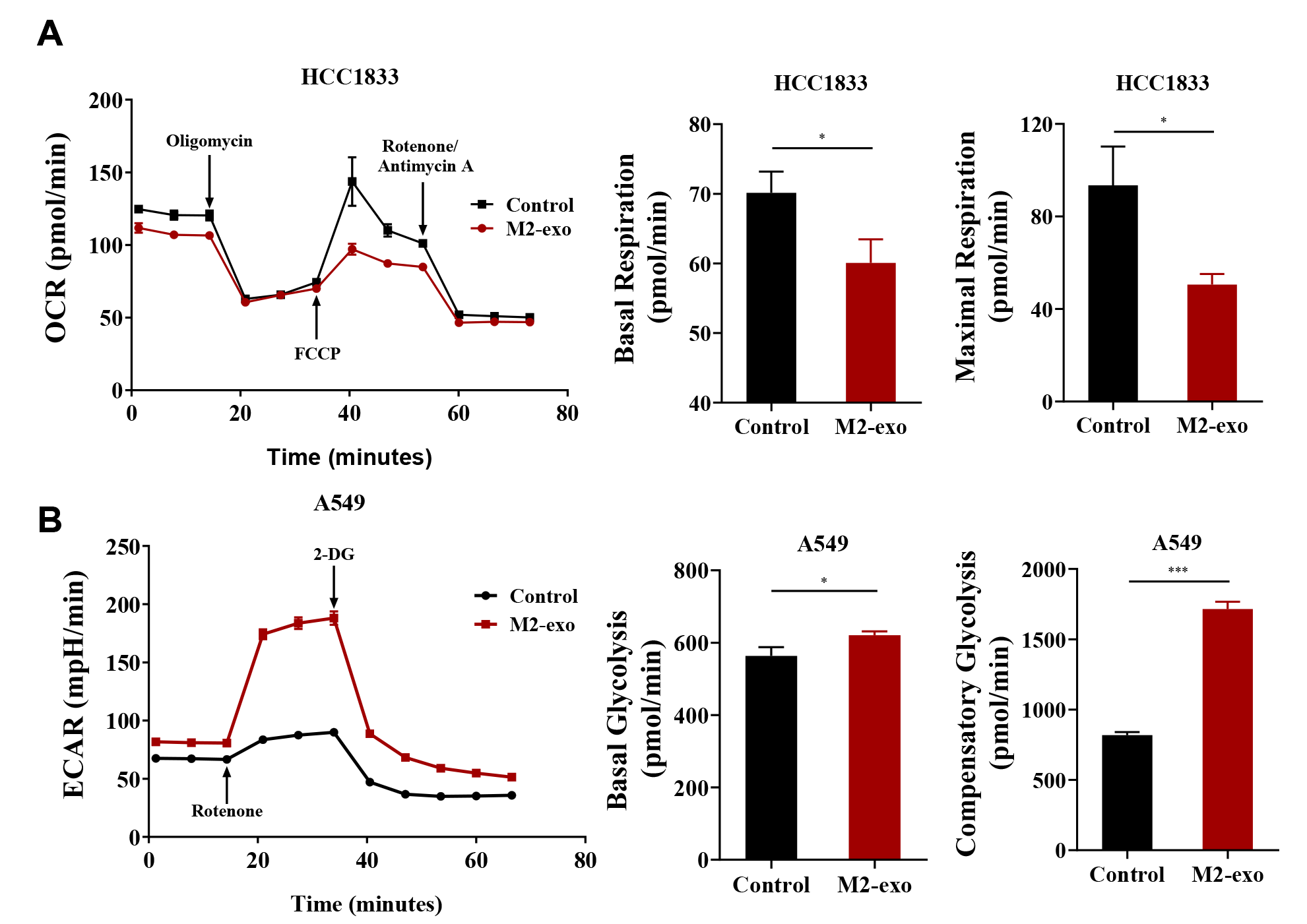


Figure S3.Effects of shcircFTO-M2-exo and M2-exo on NSCLC cells. (A) The viabilities of NSCLC cells were detected by CCK8 assays after treatment with shcircFTO-M2-exo and M2-exo. (B) The migration abilities of NSCLC cells were detected by Transwell assays after treatment with shcircFTO-M2-exo and M2-exo. Values are shown as the mean ± SD of three independent experiments. * P < 0.05; ** P < 0.01; *** P < 0.001.


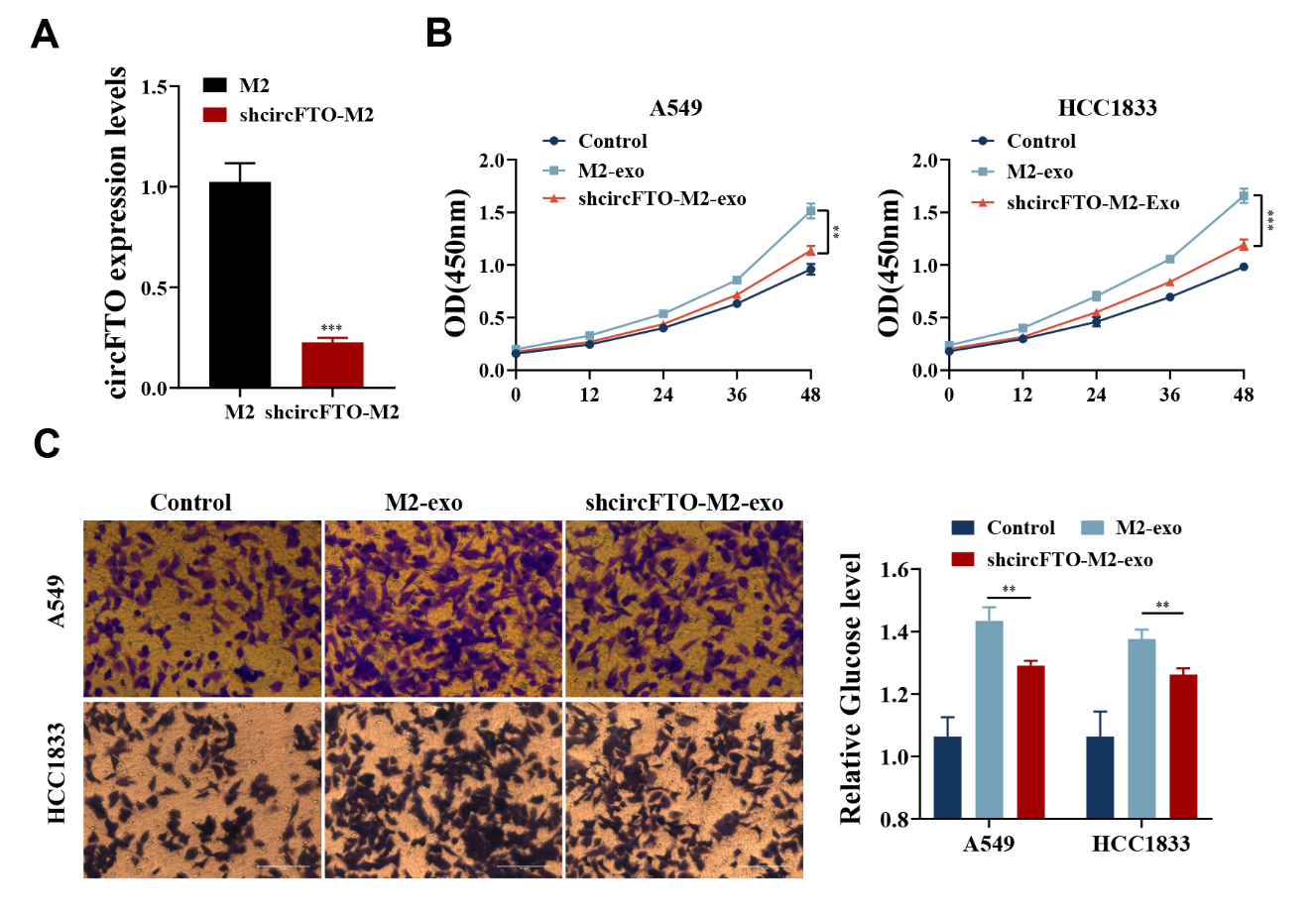


Figure S4. (A) Efficiency of circFTO-knockdown was examined by RT-qPCR. (B) KEGG pathway enrichment analysis.


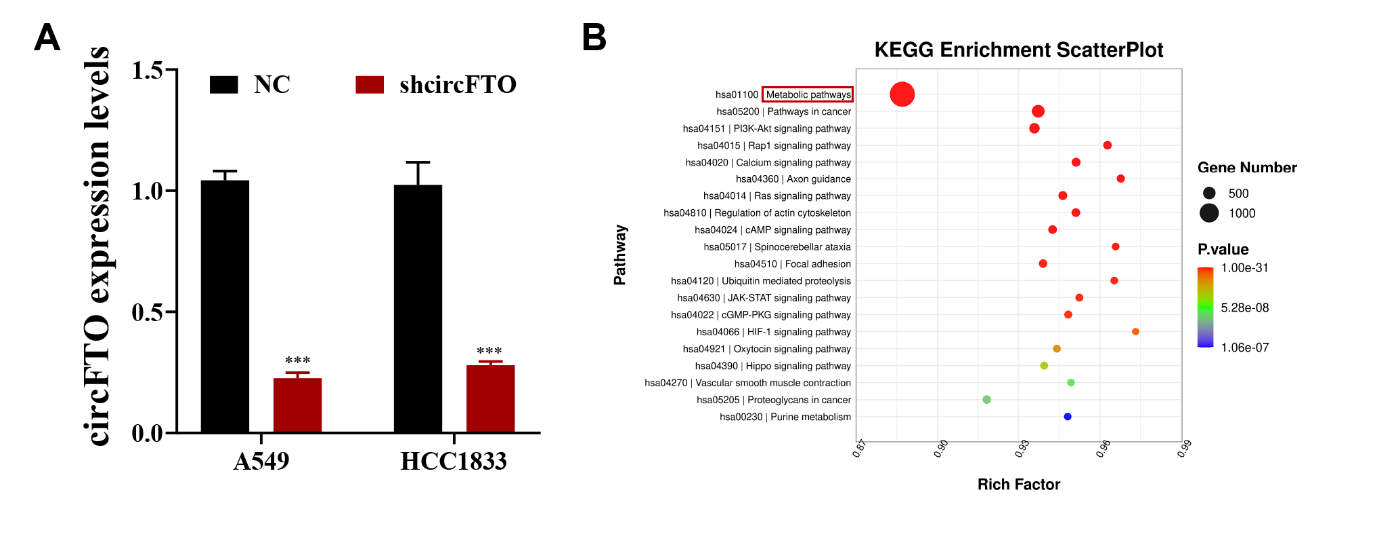


Figure S5.Glycolysis-related tests in NSCLC cells after treatment with shcircFTO-M2-exo and M2-exo. (A-C) Changes in relative glucose uptake, ATP levels, and lactate production after treatment with shcircFTO-M2-exo and M2-exo. (D) Changes in OCR and the basal/maximal respiration of HCC1833 cells after treatment with shcircFTO-M2-exo and M2-exo. (E) Changes in ECAR and glycolysis levels in A549 cells after treatment with shcircFTO-M2-exo and M2-exo.


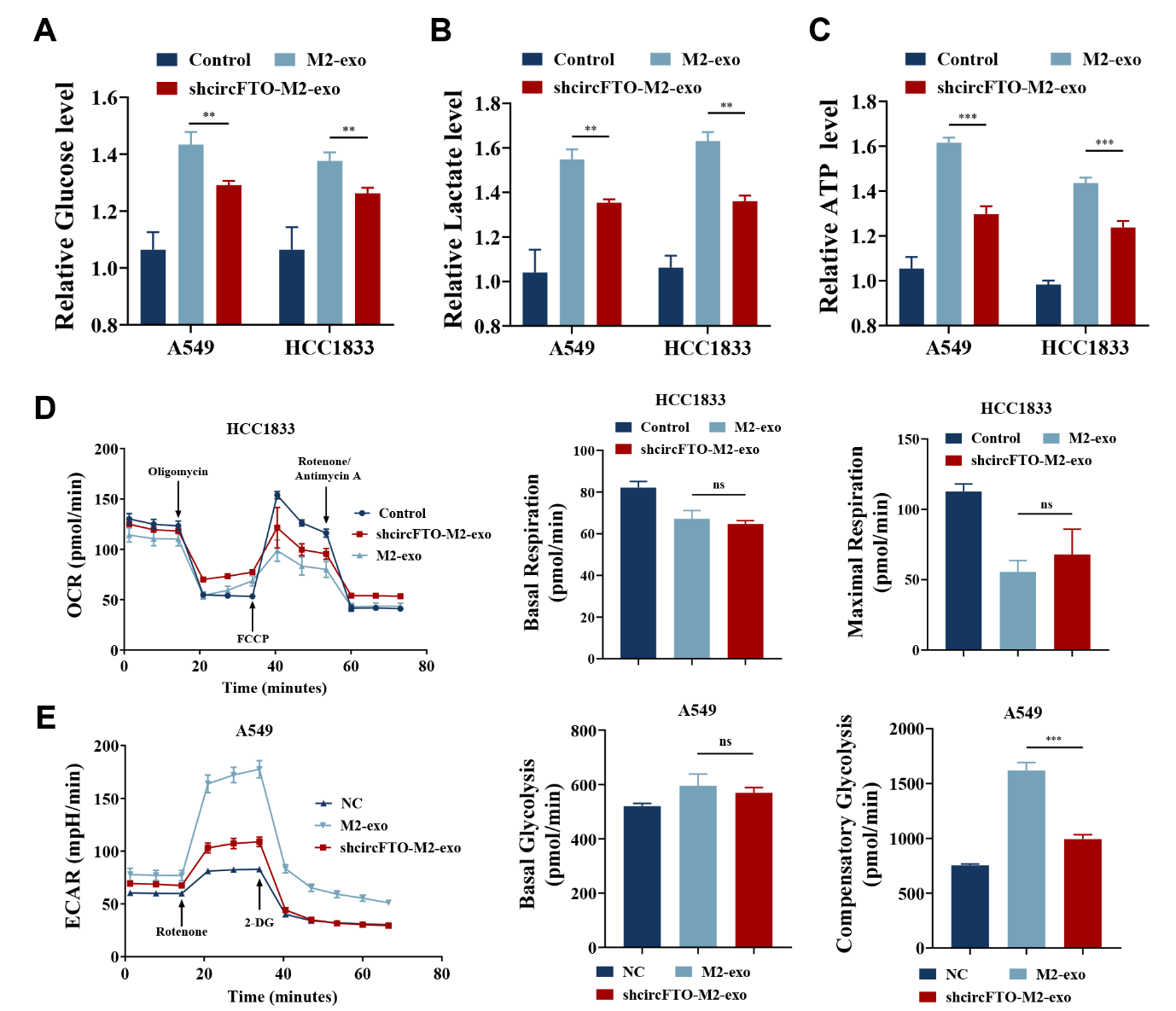


**Table S1**. Primer sequences used in this study

| Primers used for qPCR: | |
| --- | --- |
| CD206 forward | CATTAAGCAAAATCACCAGGACA |
| CD206 reverse | TACCCAGCGTGACATGAACT |
| β-actin forward | CCTGTACGCCAACACAGTGC |
| β-actin reverse | ATACTCCTGCTTGCTGATCC |
| hsa_circ_0072391 forward | AGACCGCTGCTATTCTGTCT |
| hsa_circ_0072391 reverse | TCATTCAGCAACATCCGAGC |
| hsa_circ_0005941 forward | TCGGTGGGTGGAACTAAA |
| hsa_circ_0005941 reverse | GTGGAAGAAGATGGAGGGT |
| hsa_circ_0005567 forward | TGGCAATCTCTTCTCTGAAAGCTGA |
| hsa_circ_0005567 reverse | CTCAGCTCTTCTCTAGCTTTTGCCA |
| hsa_circ_0005556 forward | GATGGACTGGTTCGCTTGGT |
| hsa_circ_0005556 reverse | TTTCGTGATGATAAAGGATGCA |
| hsa_circ_0001839 forward | AGGAGGCTGGAGAATTCA |
| hsa_circ_0001839 reverse | AATCAATGTCATCTTGTGGC |
| FTO forward | ACTTGGCTCCCTTATCTGACC |
| FTO reverse | TGTGCAGTGTGAGAAAGGCTT |
| miR-148a-3p forward | AGCAGTTCAGTGCACTACAG |
| miR-148a-3p reverse | GCAGGGTCCGAGGTATTC |
| GAPDH forward | CATCAAGAAGGTGGTGAAGC |
| GAPDH reverse | TGACAAAGTGGTCGTTGAGG |
| PDK4 forward | AGGTGGAGCATTTCTCGCGCTA |
| PDK4 reverse | GAATGTTGGCGAGTCTCACAGG |

Table S2: Correlation between clinicopathological features of NSCLC and expression of circFTO (n=80)

| Characteristics | circFTO expression |  | P Value |
| --- | --- | --- | --- |
|  | Low expression  (*n*=40) | High expression  (*n*=40) |  |
| Gender |  |  | 0.488 |
| Male | 13 | 17 |  |
| Female | 27 | 23 |  |
| Age |  |  | 0.2324 |
| ≤65 | 24 | 30 |  |
| ≥65  TNM | 16 | 10 | 0.0368 |
| Ⅰ+Ⅱ  Ⅲ+Ⅳ  Pathological grade  1+2  3+4  Tumor Diameter  ＜3cm  ＞3cm | 30  10  28  12  19  21 | 20  20  16  24  11  29 | 0.0129  0.1053 |
